# Evaluation of Copan FecalSwab as Specimen Type for Use in Xpert C. difficile Assay

**DOI:** 10.1128/JCM.00369-17

**Published:** 2017-09-25

**Authors:** Michael J. Mashock, Matthew L. Faron, Blake W. Buchan, Nathan A. Ledeboer

**Affiliations:** a Medical College of Wisconsin, Milwaukee, Wisconsin, U.S.A. America; b Wisconsin Diagnostic Laboratories, Milwaukee, Wisconsin, U.S.A; Virginia Commonwealth University Medical Center

**Keywords:** C. difficile, FecalSwab, liquid-based microbiology

## Abstract

Liquid-based microbiology (LBM) devices incorporating flocked swabs and preservation medium ease transport of specimens and improve specimen yield compared to traditional fiber wound swabs; however, the performance of LBM collection devices has not been evaluated in many molecular assays. It is unclear how the differences in matrix and specimen loading with an LBM device will affect test performance compared to traditional collection devices. The purpose of this study was to evaluate the performance of specimens collected in FecalSwab transport medium (Copan Diagnostics, Murrieta, CA) compared to unpreserved stool using the Cepheid Xpert C. difficile assay (Cepheid, Sunnyvale, CA). Results equivalent to unpreserved stool samples were obtained when 400 μl of FecalSwab-preserved stool was employed in the Xpert assay. The positive and negative percent agreement of specimens inoculated with FecalSwab medium (*n* = 281) was 97.0% (95% confidence interval [CI], 90.9 to 96.4%) and 99.4% (95% CI, 96.4 to 99.9%), respectively, compared to reference results obtained using unpreserved stool. Throughout this study, only four discrepant results occurred when comparing preserved specimens to unpreserved stool specimens in the Xpert C. difficile PCR assay. Post discrepant analysis, using the BD MAX Cdiff assay, the specificity and sensitivity both increased to 100%. The high positive and negative percent agreements observed in this study suggest that stool preserved in FecalSwab media yields equivalent results to using unpreserved stool when tested on the Xpert C. difficile assay, allowing laboratories to adopt this liquid-based microbiology collection device.

## INTRODUCTION

Clostridium difficile is a nosocomial pathogen that is responsible for a majority of antibiotic-associated diarrhea cases as well as cases of pseudomembranous colitis (1). C. difficile infections (CDI) are increasing in prevalence and severity of illness, with 793 estimated deaths in 1999 and more than 7,000 deaths in 2009 (2). Beginning in 2001, hospital discharge data in some regions demonstrated that up to 82% of C. difficile isolates belonged to the same strain (North American PFGE type 1) (3). Numerous outbreaks were observed in US and Canadian hospitals resulting in mortality rates of up to 6.9% (4). The persistent nature of C. difficile spores and the infection mortality rate of recent strains has caused clinicians to revise previous definition of diarrhea and stresses the need for early diagnosis of toxigenic C. difficile for implementation of isolation and/or treatment (5).

The implementation of liquid-based microbiology (LBM) collection devices that incorporate a flocked swab has been reported to improve bacterial adsorption and release of clinical material from the swab (6). Flocked swabs deposited a larger number of microorganisms on Gram stain slides in comparison with traditional swabs (6). The increased quantity of microorganisms caught and released could improve the detection rate of microorganisms and thereby increase the sensitivity of laboratory tests. The application of LBM collection devices also provides several workflow advantages for the laboratory, as demonstrated by Fontana et al., including reductions in costs, time savings for hospital staff, and reduction in patient discomfort (7).

In this study, we developed a protocol to examine the use of the FecalSwab for C. difficile PCR using the Xpert C. difficile assay (Cepheid, Sunnyvale CA). The FecalSwab is an LBM collection device that permits a single specimen to be collected and used for stool culture as well as for nucleic acid amplification tests. As stool samples in the LBM collection device are diluted into a preservative matrix, initial testing was required to determine equivalent loading amounts to match unpreserved stool inoculation. Once determined, the analytic sensitivity was calculated and followed by a comparison study of preserved stool versus unpreserved stool inoculation to determine the clinical positive and negative percent agreements of this collection device.

## RESULTS

### Determination of amount of FecalSwab medium required for equivalent inoculum.

The equivalency of the FecalSwab-preserved specimens was determined by comparing cycle threshold (*C_T_*) values of unpreserved specimens to those of various inoculum volumes of preserved specimen. Unpreserved specimens inoculated with a wound swab had an average *C_T_* value of 36.0 (Table 1). Similarly, 400 μl of preserved specimens resulted in an average *C_T_* score of 35.8. Analytical sensitivity testing and clinical comparison to unpreserved stool specimens was performed using 400 μl of preserved stool.

**TABLE 1:** Determination of analytic equivalency of the FecalSwab compared to the wound swab collection device

Used to inoculate elution reagent	First run (*C_T_*)	Second run (*C_T_*)	Third run (*C_T_*)	Avg (*C_T_*)	SD
Pos wound swab^*a*^	34.3	35.5	38.2	36.0	2.00
Neg wound swab^*a*^	0	0	0	0	0
200 μl FS medium^*b*^	36.7	36.8	ND^*c*^	36.7	0.071
400 μl FS medium^*b*^	35.7	35.4	36.3	35.8	0.461
800 μl FS medium^*b*^	34.4	36.7	36.3	35.8	1.23
1,000 μl FS medium^*b*^	34.0	34.8	34.6	34.5	0.421

a Positive (pos) and negative (neg) wound fiber cotton swabs were tested to determine spiked cycle threshold (*C_T_*) amount following FDA-cleared protocol.

b FecalSwab (FS) medium spiked with C. difficile BAA-1875.

c ND, not detected.

### Analytical sensitivity of FecalSwab-preserved specimens.

The analytical sensitivity of preserved specimens was compared to that of unpreserved specimens using spiked stool at either 1.5 × 10^4^, 1.5 × 10^3^, or 1.5 × 10^2^ CFU/ml (Table 2). Preserved specimens displayed a similar limit of detection compared to unpreserved specimens as all three tests detected nucleic acids at 1.5 × 10^4^ CFU/ml. The spiked stool sample at a concentration of 1.5 × 10^3^ CFU/ml was positive for one of three runs for both inoculation methods.

**TABLE 2:** Analytical sensitivity of the FecalSwab transport device using 400 μl of FecalSwab media

Test condition	*toxB C_T_*^*a*^			No. of positive specimens	No. of *C_T_* detections	Total no. of specimens
	Run 1	Run 2	Run 3			
Wound swab 10^2^ CFU/ml	38.6	ND	ND	0	1	3
FecalSwab 10^2^ CFU/ml	ND	ND	ND	0	0	3
Wound swab 10^3^ CFU/ml	37.1	36.4	38.6	1	3	3
FecalSwab 10^3^ CFU/ml	36.0	39.7	ND	1	2	3
Wound swab 10^4^ CFU/ml	32.2	34.2	35.0	3	3	3
FecalSwab 10^4^ CFU/ml	33.7	35.9	37.0	3	3	3

a *C_T_*, cycle threshold; ND, not detected.

The stability of the collection device was examined by comparing unpreserved and FecalSwab preserved stool with the Xpert C diff assay over 7 days (Table 3). The raw and preserved stool specimens both resulted positive throughout the duration of the experiment when stored at 4°C. The raw stool specimens yielded a statistically lower *C_T_* value than the preserved stools at all time points (*P* value = 0.023). The *C_T_* values of the preserved stool specimens gradually increased during the first 72 h of incubation at 4°C (*C_T_* values 33.4, 34.0, 35.5, and 35.0 at 0, 24, 48, and 72 h incubation, respectively) but were not statistically different (*P* value = 0.14, 0.14, and 0.068, respectively). The *C_T_* value for preserved stool after 7 days of incubation (35.8) was significantly greater than that at time point 0 (*P* value = 0.027). No significant change was observed in the *C_T_* values of the unpreserved stool specimens at any time point within the study (29.5, 29.4, 29.5, and 29.9 at 24 h, 48 h, 72 h, and 7 days incubation, respectively; *C_T_* value 29.1 at time zero [T0]; *P* value = 0.064).

**TABLE 3:** Determination of the nucleic acid preservative capability of the FecalSwab

Preservation employment	Incubation period	Xpert C. difficile assay result	*C_T_*^*a*^	SD
Unpreserved	0 h	Positive	29.13	0.31
	24 h	Positive	29.47	0.39
	48 h	Positive	29.40	0.57
	72 h	Positive	29.50	0.73
	7 d	Positive	29.90	0.29
Preserved by FecalSwab	0 h	Positive	32.37	0.54
	24 h	Positive	33.97	0.70
	48 h	Positive	35.50	0.94
	72 h	Positive	34.97	0.31
	7d	Positive	35.80^*b*^	0.29

a *C_T_* values are averages of three experiments.

b Significant difference compared with 0 h cycle threshold, defined as a *P* value of > 0.05.

### Comparison of wound swab to FecalSwab inoculation for the Xpert C. difficile assay.

In total, 281 residual specimens sent for C. difficile testing were enrolled in the study. The performance of the FecalSwab inoculation compared to testing of unpreserved specimens is summarized in Table 4. The preserved specimens demonstrated a sensitivity of 97.0% and a specificity of 99.4% compared to raw stool without preservation medium. The selective enrollment positive and negative percent agreements (*n* = 178) were 96.6% and 100%, respectively, while the all-comer enrollment sensitivity and specificity (*n* = 103, 11.7% positivity) was 100% and 98.9%, respectively.

**TABLE 4:** Clinical evaluation of the Xpert C. difficile assay using the FecalSwab transport device compared to the wound swab^*a*^

Enrollment	TP	TN	FP	FN	Total	PPA (95% CI)	NPA (95% CI)
Selective^*b*^	86	89	0	3	178	96.6 (91–99)	100 (96–100)
Prospective^*c*^	12	90	1	0	103	100 (70–100)	98.9 (93–100)
Total	98	179	1	3	281	97.0 (91–99)	99.4 (96–100)

a TP, true positive; TN, true negative; FP, false positive; FN, false negative; PPA, positive percent agreement; NPA, negative percent agreement, CI, confidence interval.

b Equal amounts of positive and negative cultures were enrolled. All testing was performed blinded by a separate study member.

c To ensure that selective enrollment did not affect results, all specimens ordered for C. difficile testing were enrolled and tested.

Variability in specimen enrollment was measured by comparing the change in *C_T_* values between preserved and unpreserved specimens. A Δ*C_T_* score of <1.0 was considered equivalent (8) while a Δ*C_T_* score ≥3.3 was considered to be a log difference in starting material (9). The average Δ*C_T_* value between the preserved and unpreserved specimens was 0.26; 16.7% of samples had a Δ*C_T_* value under 1.0, 16.0% of samples were at >1.0 and <3.3, and 3.2% of samples were >3.3. Use of preserved specimen resulted in a low error rate; only a single specimen was invalid and upon retesting the specimen resulted negative, yielding a first-run success rate of 99.6% (280/281 specimens).

In total, 1 false-positive (FP) and 3 false-negative (FN) specimens were observed with preserved specimens in comparison to unpreserved specimens (Table 5). Discrepant specimens were resolved using the BD Max Cdiff assay and all three false negatives were confirmed negative and the FP resulted as positive. Post discrepant resolution of the sensitivity and specificity of the preserved media was 100%. Interestingly, 3 of the 4 discrepant specimens detected the presence of *toxB* nucleic acids, but were above the cutoff threshold for the software.

**TABLE 5:** Discrepant results of wound swab and FecalSwab results, resolved via BD Max Cdiff assay

Specimen no.	Consistency	Result (*toxB* [*C_T_*])		
		Wound swab^*a*^	FecalSwab^*a*^	BD Max^*c*^
MCW_042	Thick liquid, mucoid	Positive (36.2)	Negative (n/a)	Negative
MCW_138^*b*^	Semisolid, mucoid	Positive (34.5)	Negative (38.5)	Negative
MCW_173	Liquid	Positive (36.1)	Negative (37.9)	Negative
MCW_217	Soft	Negative (37.3)	Positive (35.9)	Positive

a Performed with the Cepheid GeneXpert system using Xpert C. difficile cartridges. A threshold of 37.0 was considered a positive result. n/a, not applicable.

b Testing of FecalSwab was delayed for 60 min after Xpert cartridge was set up, which is outside parameters recommended by the package insert instructions.

c Performed with the BD Max Cdiff assay as a molecular test to rectify discrepant results.

## DISCUSSION

The potential of nosocomial C. difficile infections to spread throughout a hospital, combined with the potentially severe nature of these infections, make timely and efficient detection of toxigenic C. difficile strains critical. Nontoxic strains of C. difficile frequently colonize patients but do not cause illness, thus the presence of C. difficile nucleic acids does not necessarily indicate a disease state. As such, numerous strategies have evolved in clinical laboratories to detect toxic strains of C. difficile and only treat those that fit criteria for pathogenic infections (10, 11). Some clinicians prefer a multistep approach in which the presence of toxins or glutamate dehydrogenase is initially detected, followed by nucleic acid amplification tests (NAAT) for C. difficile to avoid unnecessary treatment of colonized individuals. Alternatively, other laboratories prefer to use a single NAAT to identify all individuals infected with C. difficile and then use patient history to determine if treatment is appropriate. A study by Barbut et al. observed positive impacts on patient care using either strategy, a prescreening step plus NAAT or a standalone NAAT, to rapidly diagnosis C. difficile infections (10).

The previous gold standard to identify C. difficile was toxigenic culture, but enzyme immunoassays are still frequently employed to detect C. difficile toxin production with less turnaround time (TAT). However, enzyme immunoassays display low sensitivity (53 to 61%) compared with nucleic acid amplification tests (96 to 98%) (12, 13). The high sensitivity and specificity of molecular approaches for accurate and rapid detection of CDI were previously examined (14) and the Xpert C. difficile assay displayed high sensitivity (100%) and a high specificity (91.7%) with less than 2 h turnaround time.

Throughout this study, 400 μl of preserved specimen displayed equivalent *C_T_* values to unpreserved specimens in the Xpert C. difficile assay, while 1,000-μl additions displayed lower *C_T_* scores compared to 400-μl additions (Table 1). Feces is a difficult matrix to work with, as it has varied viscosity, high particulate content, and the presence of inhibitory substances, any of which can lead to indeterminate results (14). As such, when working with unpreserved stool overloading of the wound fiber swab should be avoided (15) as excess fecal material can result in an inhibitory effect with the Xpert C. difficile assay (package insert). The lower *C_T_* score observed in the stability assay for the FecalSwab preserved stool could be due to dilution of the spiked sample.

There were limitations within this study. The Xpert C. difficile assay used for this study does not test for the BI/NAP1/O27 strain type and thus testing only revealed the presence of one species of toxin-producing C. difficile. Sample preparation for the FecalSwab requires diluting the specimen in the storage medium, which may have reduced the quantity of nucleic acids and thus resulted in the false negatives observed. Another limitation is associated with discrepant result analysis, as all discrepant samples were cryogenically preserved at −80°C. All discrepant specimens required a freeze/thaw cycle prior to analysis using the BD Max Cdiff assay. The freezing and thawing of samples could have reduced stability of the specimens and otherwise altered the discrepant results.

A further limitation of this study involves the limit of detection of the Xpert C. difficile assay, as this was determined using a single C. difficile-spiked negative stool specimen. These spiked samples were not plated to confirm bacterial counts, as normal flora found within the negative stool would outnumber C. difficile counts and make bacterial counts uninterpretable. Without bacterial counts the initial bacterial concentrations employed to calculate the limit of detection could have been incorrect, resulting in less-than-accurate determination of the limit of detection (LOD).

This study was undertaken to evaluate the use of preserved stool specimens for molecular testing of toxigenic C. difficile. The FecalSwab collection device has displayed improved preservative properties compared to those of other collection devices. A comparison of the Eswab and the FecalSwab at 4°C for 48 h, as well as after 2 weeks of storage at −20°C, resulted in equal preservation of diarrheagenic bacterial species between the two collection devices (16).

The high sensitivity and specificity of the Copan FecalSwab-preserved specimens observed within this study compared with those of the unpreserved specimens suggest that this preservation medium is suitable for collection of samples for use in the Xpert C. difficile assay.

## MATERIALS AND METHODS

### Specimen loading equivalency and analytical sensitivity.

A 0.5 McFarland suspension of C. difficile strain BAA-1875 (approximately 1.5 × 10^7^ CFU/ml) was created in saline solution and 0.5 ml was added to 4.5 ml of a single negative stool (clinical sample confirmed negative by Xpert Cdiff assay) and vortexed thoroughly. The FecalSwab was inoculated by dipping the flocked swab into the contrived sample, covering the entire swab, and then placing the swab into the included transport medium, followed by breaking off the swab and securing the cap. Xpert elution reagent was then inoculated with 200 μl, 400 μl, 800 μl, or 1,000 μl of FecalSwab preservation medium or with diluted stool as per the Xpert package insert. Testing was performed in triplicate for each volume tested. Xpert elution reagents were vortexed and added to cartridges following the package insert. The amount of preservation medium that was equivalent to results obtained using the unpreserved specimen was used for further testing.

To determine analytical sensitivity, a 0.5 McFarland suspension was generated in saline using strain BAA-1875 and serial 10-fold dilutions were performed to obtain concentrations of C. difficile at 1.5 × 10^5^ to 1.5 × 10^3^ CFU/ml. An aliquot of 200 μl for each bacterial concentration was added to 1,800 μl of a single negative stool (as per Xpert testing) and vortexed thoroughly to create a final concentration ranging from 1.5 × 10^4^ to 1.5 × 10^2^ CFU/ml (no culture was performed). Xpert elution reagent vials were inoculated in triplicate with each concentration, with 400 μl of either preserved or unpreserved specimen. The lowest concentration at which at least two of the three replicates resulted positive was defined as the limit of detection.

### Specimen enrollment.

Residual unpreserved stool specimens submitted for C. difficile PCR were enrolled into the study after standard of care testing was completed from February to June 2016. There was less than a 24 h delay between clinical standard of care testing and testing using the FecalSwab and specimens were stored at 4°C during the interim. Only liquid or unformed stool specimens that assumed the shape of the storage container were enrolled. Specimens were excluded if standard of care testing was performed >24 h from specimen enrollment. Only one specimen per patient was enrolled for testing and an aliquot of unpreserved stool was saved for each specimen at −80°C for future discrepant testing. Specimen enrollment in this study involved two stages: “selected enrollment” for equal amounts of positive and negative specimens (*n* = 178) or “prospective enrollment” of any unformed stool specimen submitted for C. difficile PCR testing received within the laboratory (*n* = 103). Specimens for this study were enrolled following criteria established by the Medical College of Wisconsin Institutional Review Board (MCW IRB)-approved protocol.

### Standard of care testing.

Specimens were tested using the Cepheid Xpert C. difficile assay in accordance with the manufacturer's package insert. Briefly, wound cotton fiber swabs were inoculated with unpreserved fecal specimen and broken off into the Xpert elution reagent vial. The elution reagent vial was vortexed at high speed for 10 s and the entire volume subsequently transferred to the “S” chamber of the Xpert C. difficile assay cartridge using a transfer pipette. Results from the Xpert test were recorded for each specimen.

### FecalSwab testing.

Residual clinical specimens meeting the MCW IRB-approved protocol inclusion criteria were enrolled and deidentified prior to specimen loading into FecalSwab preservation medium. Briefly, the flocked swab from the FecalSwab collection kit was saturated with stool specimen to inoculate with at least 200 mg stool (weighed on scale to confirm) and then returned to the screwcap tube and vortexed at high speed for 10 s. A total of 400 μl of preservation medium was added to the Xpert elution reagent vial and vortexed at high speed for 10 s. The entire volume was pipetted into the “S” chamber of the Xpert C. difficile assay cartridge and loaded into the GX16 instrument.

### Determining stability of FecalSwab for C. difficile testing.

The stability of C. difficile specimens inoculated into the FecalSwab was measured by analyzing pooled C. difficile-spiked stool on the Xpert C. difficile assay with or without preservation over 7 days and comparing C_*T*_ scores to those at time point 0. Stool specimens from four separate Xpert-negative patients were deidentified and enrolled as per MCW IRB-approved protocol and pooled to use as the sample matrix for spiking. C. difficile ATCC 1875 strain was used to create a 0.5 McFarland in saline solution (∼1.5 × 10^7^ CFU/ml). The 0.5 McFarland solution was diluted 100-fold in saline and spiked into the stool to reach a final concentration of 1.5 × 10^5^ CFU/ml, approximately 3× the limit of detection. The spiked stool was vortexed thoroughly to mix prior to inoculation of 18 FecalSwabs following manufacturer's protocol. All collection devices were thoroughly vortexed after spiking and all but three FecalSwabs were immediately incubated at 4°C. Three spiked FecalSwabs were tested on the Xpert C. difficile assay and defined as time point 0 to establish an initial cycle threshold score for the preserved spiked stool. The remaining unpreserved stool was also tested in triplicate to establish an initial cycle threshold score for the unpreserved spiked stool. The remaining raw spiked stool was stored at 4°C. Preserved and unpreserved stool were then tested in triplicate at the following time points: 24 h, 48 h, 72 h, and 7 days.

### Discordant analysis.

Discordant results were analyzed using the BD Max Cdiff assay (BD diagnostics, Franklin Lakes, NJ) as per manufacturer protocol. Specimens were removed from −80°C storage and allowed to thaw at room temperature for a total of 1 freeze-thaw cycle. A disposable 10-μl inoculation loop was employed to retrieve fecal specimen, as per package insert, and dispersed into the sample buffer tube by vigorously twirling the inoculation loop. The inoculated sample buffer tube was then vortexed at high speed for 1 min and the sample buffer tube, BD Max Cdiff reagent strip, and BD Max Cdiff PCR cartridge were loaded onto the BD Max system and tested.

### Statistical analysis.

GeneXpert results for FecalSwab-preserved stool specimens were compared to results from unpreserved specimens. The positive percent agreement and negative percent agreement of the preserved specimens was calculated as per FDA guidance (17). Positive percent agreement was defined as the number of positive results concordant between the two methods divided by the total concordant and false negative, multiplied by 100. Negative percent agreement was defined as the number of negative results concordant between the two methods divided by the total concordant and false positive, multiplied by 100. Significant difference was calculated using Student's *t* test, with a *P* value < 0.05 being considered statistically significant.
